# Poverty as Biography in Motion: Agency, Emotion, and the Limits of Contemporary Poverty Theory

**DOI:** 10.1111/1468-4446.70127

**Published:** 2026-05-17

**Authors:** Suzanne Butler

**Affiliations:** ^1^ Department of Geography Newcastle University Politics and Sociology Newcastle upon Tyne UK

## Abstract

Despite extensive research, poverty remains theoretically under‐explained. Dominant approaches oscillate between structural, resource‐based models that conceptualise poverty as a measurable condition, and behavioural or responsibilisation accounts that locate persistence in individual conduct. Whilst often treated as opposing theses, this article argues that both rest on a shared theoretical error: the mis‐specification of agency as either absent or over‐moralised. As a result, poverty is rendered static, episodic, or behaviourally deficient, limiting the capacity of theory to explain endurance, stalling, and fragile change across lives. The article advances a different ontological starting point by reconceptualising poverty as a temporal, emotional, and biographical process. Drawing on feminist sociology, life‐course theory, and narrative perspectives, it introduces the concept of *poverty as biography in motion*, alongside a trajectory‐based analytic that theorises turbulence, stalling, and fragile progress as patterned temporal forms of constraint. Central to this framework is the treatment of emotion as a mediating mechanism through which structural conditions are lived, and agency is exercised over time. By reframing agency as situated, relational, and emotionally mediated, the article moves beyond entrenched binaries between structure and behaviour. It offers a theoretical grammar capable of explaining poverty's persistence and uneven movement without reverting to static or moralised accounts. In doing so, the article contributes to poverty theory by reorienting explanation toward lived process, duration, and mediation, and by foregrounding biography as a central site of sociological analysis.

## 1 Introduction

Despite decades of empirical and policy‐focused research, poverty remains theoretically unsettled. Sociological and social policy scholarship has produced increasingly sophisticated accounts of deprivation, inequality, and exclusion, yet it has been less successful in explaining how poverty is lived, sustained, and, at times, unevenly reconfigured across the life course. In particular, whilst academic poverty research has been dominated by structural and resource‐based explanations, these exist alongside—and are often implicitly shaped by—powerful policy and public discourses that foreground individual responsibility, behaviour, and moral judgement. The relationship between these domains is not symmetrical, but it is analytically significant. Together, they structure how poverty is explained, debated, and experienced, whilst leaving key aspects of agency under‐theorised.

This article argues that a central limitation of contemporary poverty theory lies not in a simple division between competing schools, but in a shared difficulty in adequately conceptualising agency across these domains. Within academic scholarship, agency is often attenuated, appearing only indirectly within accounts focused on distribution, resources, and structural constraint (Lister 2004). In policy and public discourse, by contrast, agency is highly visible but moralised, framed through narratives of responsibility, choice, and behavioural correction (as discussed in Patrick 2017b; O’Hara 2020). Whilst these framings differ in emphasis and intent, they converge in narrowing what counts as meaningful action. Agency is either backgrounded or judged, rather than understood as a situated, bounded and evolving capacity exercised within constraint. As a result, poverty is difficult to theorise as something lived across time: as endurance, adaptation, or fragile and reversible change shaped through ongoing interaction between structure, agency, and emotional–relational dynamics.

At the same time, contemporary poverty scholarship is not reducible to these limitations. A substantial and growing body of qualitative and longitudinal research has sought to capture the lived experience of poverty, emphasising its temporal, relational, and affective dimensions (Millar 2007; Shildrick and MacDonald 2013; Patrick 2016a; Dermott and Pantazis 2014). This work has demonstrated that poverty is rarely experienced as a discrete episode, but as a cumulative and often uncertain process unfolding across lives. It has illuminated the everyday practices through which people manage scarcity, sustain care, and navigate stigma, as well as the emotional and relational dynamics through which poverty is felt, interpreted, and negotiated (Reay 1996a; Walker 2014). However, whilst these insights have been empirically rich, they have often remained theoretically dispersed. They tend to sit alongside dominant structural frameworks, or in critique of policy discourses, rather than being fully integrated into a re‐specification of agency within poverty theory itself.

This article builds on and extends this body of work by offering a more explicit theoretical reconstruction. It argues that understanding poverty requires moving beyond static or event‐based conceptualisations and toward an account that treats lives themselves as the primary unit of analysis. Rather than approaching poverty as a condition to be measured or a behaviour to be corrected, the article conceptualises it as a temporal, emotional–relational, and biographical process. In doing so, it introduces the concept of *poverty as biography in motion*: a framework that foregrounds how structural conditions are lived through time, how agency is exercised within constraint, and how emotional–relational dynamics mediate what forms of action become possible across the life course.

This intervention is situated in dialog with, rather than in opposition to, existing traditions. The relative deprivation approach, most clearly articulated by Townsend (1979) and extended through subsequent empirical studies (Mack and Lansley 1985; Gordon et al. 2000), has been foundational in establishing poverty as socially produced and structurally patterned. At the same time, its analytical focus on measurement, thresholds, and distribution has tended to render agency implicit and time as duration rather than lived process. Conversely, behavioural and responsibilisation framings—most visible within policy discourse but also shaping broader understandings of poverty—foreground agency but do so in normatively constrained ways, equating action with moral deficiency, responsibilisation and blame (as discussed in Welshman 2013; Patrick 2016a; O’Hara 2020). Whilst these approaches differ in emphasis, both encounter difficulty in explaining forms of poverty characterised by endurance without improvement, fragile and reversible change, or prolonged periods of apparent stasis that nonetheless involve ongoing effort, judgement, and constraint.

The argument developed here is therefore not that existing frameworks are wholly inadequate, but that they are limited by a shared mis‐specification of agency and temporality. By treating action either as attenuated or as overly individualised, they obscure how poverty is actively lived across time, and how structural conditions are mediated through emotional–relational dynamics that shape judgement, risk, and possibility. Addressing this limitation requires a shift in theoretical starting point: from status or behaviour to biography; from events to lived duration; and from emotion as outcome to emotion as part of the causal processes through which poverty is sustained and, at times, reconfigured.

To develop this shift, the article makes three linked theoretical contributions. First, it reconceptualises poverty as a biographical and processual phenomenon, foregrounding time, continuity, and lived experience rather than static or status condition. Second, it introduces a trajectory‐based analytic—turbulence, stalling, and fragile progress—to capture patterned temporal forms through which poverty unfolds across lives. Third, it theorises emotional–relational dynamics as mediating mechanisms through which structural conditions are interpreted and acted upon, linking macro‐level constraints to lived outcomes without reducing agency to either absence or deficient individual choice.

The article proceeds as follows. The next section examines dominant structural approaches within poverty scholarship alongside behavioural and responsibilisation framings that circulate within policy and public discourse, showing how each, in different ways, constrains the conceptualisation of agency. It then considers the theoretical consequences of this reduction, particularly in relation to how poverty is conceptualised as status, how time is treated as event, and how emotion is positioned as outcome rather than mechanism. The article subsequently develops an alternative ontological framing that centres biography, temporality, and emotional–relational mediation, before elaborating the concept of poverty as biography in motion and its associated trajectory‐based analytic. In doing so, it contributes not only to poverty theory, but to broader sociological debates on agency, temporality, and the relationship between structure and lived experience. By following lives through time, the article argues, poverty can be more adequately understood not simply as a condition or outcome, but as a dynamic and uneven process through which inequality is lived, negotiated, and, at times, partially transformed.

## 2 Poverty Theory's False Choice: Structure or Behaviour?

Contemporary poverty scholarship is characterised by a dominant structural tradition that exists alongside—and is often implicitly shaped in dialog with—policy and public discourses that foreground behavioural responsibility. Whilst these domains are analytically and ideologically distinct, they are not independent. Together, they structure how poverty is explained, interpreted, and responded to. This section argues that, despite their different emphases, both domains rely on restricted conceptualisations of agency that limit their capacity to explain how poverty is lived and reproduced across time.

### 2.1 Relative Deprivation and the Disappearance of Agency

Within academic poverty scholarship, structural and resource‐based approaches have been central. Townsend's (1979) reconceptualisation of poverty as exclusion from customary social participation marked a decisive shift away from subsistence‐based definitions and established poverty as socially and historically situated. Subsequent empirical developments, including Breadline Britain and the Poverty and Social Exclusion studies, have refined this approach through the operationalisation of deprivation as enforced lack, enabling detailed mapping of poverty's scale, depth, and distribution (Mack and Lansley 1985; Gordon et al. 2000). More recent work has extended these insights through increasingly sophisticated multidimensional measures of disadvantage (Levitas et al., 2007; Bradshaw et al. 2017; Edmiston 2022).

These contributions have been indispensable in demonstrating that poverty is structurally produced and socially patterned. They have provided a robust empirical foundation for challenging individualising accounts and for informing policy. However, their analytical orientation remains focused on classification, distribution, and incidence. Poverty is primarily conceptualised as a condition that individuals or households occupy, measured at particular points in time or across durations. Whilst such approaches can identify persistence and recurrence, they tend to treat time as exposure length rather than as lived process.

Within this framework, agency is present but analytically attenuated. Individuals are positioned primarily in relation to structural conditions, with limited attention to how everyday judgements, adaptations, and forms of labour shape how poverty is navigated across lives. Where agency is operationalised, it is often through observable transitions—such as movements into employment or changes in household composition—rather than as an ongoing, situated capacity exercised within constraint (Millar 2007). As a result, structural approaches are highly effective at identifying who is poor and for how long, but less well equipped to explain how poverty is lived across biographies, or why similar structural conditions may produce divergent temporal trajectories.

This limitation is particularly evident in accounts of long‐term or recurrent poverty. Whilst persistence can be identified statistically, there is less analytic traction on how poverty is experienced as repetition, stalling, or fragile progress. Periods of apparent stasis—where little measurable change occurs—are difficult to theorise, even though they often involve sustained effort, adaptation, and constraint (Shildrick and MacDonald 2013). In this sense, structural approaches succeed in mapping inequality but remain less able to account for its lived reproduction over time.

### 2.2 Behavioural Accounts and the Inflation of Agency

In contrast, behavioural and responsibilisation accounts operate most prominently within policy and public discourse, although they also shape broader understandings of poverty. Drawing on a longer lineage of thought, including underclass theories that emphasised cultural and behavioural explanations (Murray, 1984), these framings have been rearticulated in contemporary welfare regimes through an emphasis on activation, conditionality, and behavioural change (Patrick 2016a; O’Hara 2020; Welshman 2013).

Within these discourses, poverty is frequently framed in terms of individual responsibility, motivation, and conduct. Agency is therefore highly visible, but also normatively specified. Individuals are positioned as decision‐makers whose actions—regarding work, family life, or engagement with welfare systems—are expected to align with particular trajectories of self‐sufficiency. Where poverty persists, it is often explained through reference to behavioural deficiencies, weak motivation, or poor decision‐making, with policy responses oriented toward correcting or incentivising conduct.

A substantial body of sociological research has critiqued these framings, demonstrating how they individualise responsibility whilst obscuring structural constraint, and how they contribute to the stigmatisation and moral regulation of those in poverty (Shildrick et al., 2012; Patrick 2016a; Walker 2014). However, their significance extends beyond their normative implications. These discourses also shape how agency is implicitly theorised, not only in policy but in the broader conceptual landscape within which poverty research operates.

By equating agency with choice, compliance, or rational decision‐making, behavioural framings detach action from the conditions under which it is exercised. They tend to assume that options are equally available, risks equally calculable, and futures equally imaginable. Emotional–relational dynamics—such as fear, shame, or exhaustion—are marginalised or reframed as attitudinal barriers rather than understood as socially patterned responses to insecurity and judgement (Walker 2014). Forms of action oriented toward stability, including risk aversion, conservation of resources, or prioritisation of care, may be recast as passivity or lack of aspiration. In this way, agency becomes visible only when it aligns with expected trajectories of exit, whilst other forms of action are rendered analytically insignificant or morally suspect.

### 2.3 A Shared Reduction: Agency as Choice or Absence

Despite their differences, relative deprivation and behavioural frameworks converge in their treatment of agency. In structural models, agency recedes into the background, overshadowed by distributions and thresholds. In behavioural accounts, it is foregrounded but stripped of context, reduced to individual decision‐making and moral compliance. In both cases, agency is flattened into a binary: present or absent, exercised correctly or incorrectly. What is missing is a conception of agency as situated, relational, and temporally extended—One that recognises how people act within constraint, how they manage risk and uncertainty, and how emotional dynamics shape what action feels possible at particular moments. This shared reduction has significant theoretical consequences. It encourages static or episodic accounts of poverty, where lives are understood through snapshots or transitions rather than through ongoing processes. It obscures the significance of endurance, stalling, and fragile progress, rendering them analytically invisible or normatively devalued. It also reinforces moralised distinctions between movement and stasis, effort and failure, which echo public discourses of deservingness and responsibility (Skeggs 1997; Patrick 2017).

Feminist poverty scholarship has long highlighted these limitations. Analyses of women's poverty demonstrate how care responsibilities, household dynamics, and welfare regimes produce forms of constraint that cannot be captured by models focused solely on income or behaviour (Glendinning and Millar 1987; Bennett 2014). Narrative and life‐course studies further show that poverty is experienced as a cumulative and relational process, shaped by timing, relationships, and emotional labour across decades rather than by isolated events (Elder 1998; Millar 2007). Yet these insights have rarely been integrated into a rethinking of agency itself. Instead, they have been treated as contextual detail, supplementing rather than challenging dominant theoretical assumptions. The result is a poverty theory that can describe inequality but struggles to explain its lived persistence. By mis‐specifying agency as either choice or absence, existing frameworks foreclose the possibility of understanding how poverty is actively lived through time—how people endure, adapt, conserve, and occasionally reorient within structural limits. Addressing this gap requires not simply adding variables or refining measures, but revisiting the ontological assumptions that underpin how poverty and agency are conceptualised.

## 3 The Theoretical Consequences of Mis‐Specifying Agency

Reducing agency to either choice or absence has consequences that extend far beyond debates about individual responsibility. It shapes how poverty itself is conceptualised, delimiting what counts as explanation, which forms of action are rendered visible, and which lives appear intelligible within sociological theory. When agency is mis‐specified in this way, poverty is inevitably theorised as a static condition or a sequence of discrete events, rather than as a lived and unfolding process. This section traces three interrelated consequences of this reduction: the reification of poverty as status rather than process, the treatment of time as event‐based rather than durational, and the relegation of emotion to outcome rather than mechanism.

### 3.1 Poverty as Status Rather Than Process

One of the most enduring consequences of agency reduction is the treatment of poverty as a condition that individuals occupy, exit, or re‐enter, rather than as a process lived across time. In relative deprivation and resource‐based frameworks, poverty is operationalised through thresholds, indicators, and durations. Although these measures can capture persistence statistically, they tend to reify poverty as a state—something one *is* at a given moment—rather than as an ongoing social relation that unfolds across biographies (Townsend 1979; Gordon et al. 2000). This status‐based framing sits comfortably alongside a thin conception of agency. If individuals are positioned primarily as recipients of structural forces, then poverty appears as an externally imposed condition, alleviated or intensified by policy change, labour market shifts, or household transitions. What happens between such moments recedes from view. The everyday work of managing scarcity, negotiating obligations, and recalibrating expectations is treated as background noise rather than as constitutive of poverty itself. Endurance becomes analytically inert; stasis appears as absence rather than as a patterned outcome of constraint.

Behavioural frameworks reproduce this status logic in a different register. Here, poverty is framed as a failure to exit a condition that is assumed to be temporary or escapable through correct action. Agency is hyper‐visible, but poverty remains static: a problem to be solved through better choices. When individuals do not exit poverty, the explanation lies not in the processual accumulation of constraint, but in deficient conduct. In both cases, poverty is theorised as something that *happens to* individuals or something they *fail to leave*, rather than as a lived trajectory shaped through ongoing interaction between structure, agency, and emotion. The result is a theoretical blind spot around forms of poverty that are neither episodic nor catastrophic, but slow, sedimented, and enduring. Long periods of “nothing happening”—no dramatic decline, no clear improvement—are difficult to render intelligible within status‐based models. Yet it is precisely within these periods that poverty is most deeply lived and reproduced, through repetition, exhaustion, and the narrowing of horizons (Shildrick and MacDonald 2013).

### 3.2 Time as Events, Not Lived Duration

A second consequence of mis‐specifying agency is the treatment of time as a sequence of events rather than as lived duration. Much poverty research, even when attentive to change, conceptualises time through transitions: entry into or exit from poverty, changes in employment, household formation or dissolution. These moments are analytically privileged because they are measurable, observable, and amenable to policy intervention (Millar 2007). Agency, correspondingly, is located at points of decision or compliance surrounding these events. Whilst such approaches have generated valuable insights, they struggle to account for how lives are shaped in the long stretches between transitions. Poverty is rarely experienced as a series of discrete moments; it is lived as continuity, repetition, and waiting. Feminist and life‐course scholars have shown that timing matters not only in relation to events, but in relation to *when* they occur within biographies, how they intersect with care responsibilities, and how their emotional consequences accumulate (Elder 1998; Millar 2007). Yet when agency is reduced to event‐based decision‐making, these temporal dimensions remain under‐theorised.

Lives characterised by prolonged constraint without dramatic deterioration or improvement. Within event‐focused frameworks, such lives appear static or uneventful, analytically thin. But from the standpoint of those living them, stalling involves constant adjustment: managing risk, conserving energy, maintaining dignity, and sustaining care under conditions of limited possibility. These forms of action are not captured by models that locate agency only at moments of transition, nor by behavioural accounts that interpret non‐movement as failure or apathy. By privileging events over duration, poverty theory also struggles to explain fragility and reversibility. Apparent “progress” may be tentative, contingent, and easily undone; moments of improvement may coexist with ongoing insecurity. Without a processual account of agency and time, such dynamics are either overstated as success or dismissed as noise. Poverty thus appears more linear, more decisive, and more responsive to intervention than it is in lived reality (Shildrick et al. 2017).

### 3.3 Emotion as Outcome Rather Than Mechanism

A third consequence is the tendency to position emotional and relational dynamics as by‐products of poverty rather than as integral to its reproduction. Across both structural and behavioural framings, emotions such as shame, fear, or exhaustion are acknowledged, but are typically treated as consequences of deprivation or stigma rather than as forces shaping action and inaction. This reflects an underlying assumption that agency is primarily cognitive or rational, whilst emotion belongs to the realm of experience rather than explanation. Yet sociological research has consistently demonstrated that emotional and relational dynamics play a central role in shaping how poverty is navigated. Shame can narrow horizons and discourage risk; fear can orient decisions toward safety rather than opportunity; exhaustion can limit the capacity to imagine or pursue change (Reay 1996b; Walker 2014). These are not simply subjective responses, but socially patterned dynamics embedded in relations of judgement, recognition, and inequality.

When such dynamics are treated as outcomes rather than mechanisms, poverty theory loses explanatory traction. Behavioural framings may reinterpret emotional responses as attitudinal deficits, whilst structural approaches may bracket them as secondary effects. In both cases, their role in shaping judgement, risk, and action is under‐theorised. This contributes to misrecognition of forms of agency that do not align with normative expectations of mobility or change. Endurance may be read as resignation; caution as lack of aspiration; withdrawal as passivity.

Reframing emotional–relational dynamics as mediating mechanisms allows for a different account. It foregrounds how structural conditions are lived and interpreted, how futures are imagined or foreclosed, and how action is oriented within constraint. In this sense, emotion is not external to agency, but constitutive of it—shaping how possibilities are evaluated and how trajectories unfold over time.

### 3.4 The Cumulative Effect: Lives Rendered Unintelligible

Ultimately these consequences produce a poverty theory that struggles to recognise the lived complexity of impoverished lives. Status‐based models flatten biographies into snapshots; event‐focused accounts privilege transitions over endurance; emotion‐blind frameworks misinterpret adaptation as failure. The cumulative effect is that large swathes of lived experience—particularly those marked by stalling, repetition, and fragile movement—remain theoretically unintelligible. This unintelligibility has normative as well as analytic implications. Lives that do not conform to expected narratives of mobility or exit are rendered deficient or invisible. Effort that does not produce improvement is discounted; forms of agency oriented toward survival, care, or dignity are devalued. In this way, theoretical mis‐specification mirrors and reinforces the moral hierarchies present in public and policy discourse, reproducing distinctions between “moving” and “stuck” lives that fail to capture how poverty is actually lived (Patrick 2017; O’Hara 2020).

Addressing these limitations requires more than refining existing models. It requires revisiting the ontological assumptions that underpin how poverty, agency, time, and emotion are conceptualised. If poverty is understood as a lived process rather than a static condition, and if agency is recognised as emotionally mediated and temporally extended, then endurance, stalling, and fragile progress become central to explanation rather than residual categories. The following section begins this task by outlining an alternative ontological starting point for theorising poverty—one that treats lives, rather than statuses or behaviours, as the primary unit of analysis.

## 4 Toward a Different Ontological Starting Point

If dominant poverty theories struggle to explain endurance, stalling, and fragile change, this is not because they lack empirical sophistication, but because they begin from an ontological framing that misrecognises how poverty is lived. When poverty is conceptualised as a status to be measured or a behaviour to be corrected, lives appear static, episodic, or deficient. What is required, instead, is a starting point that treats poverty as a process unfolding across time, mediated through relationships, institutions, and emotion. This section outlines such a shift, arguing for an ontological reframing that places lived biography—rather than condition or conduct—at the centre of poverty theory.

### 4.1 Poverty as Lived Process, Not Social Position

A processual understanding of poverty begins from the recognition that deprivation is not simply experienced at discrete moments, nor reducible to income thresholds or behavioural outcomes. Feminist and life‐course scholars have long argued that poverty is lived across biographies, shaped by cumulative exposures, timing, and relational obligations (Elder 1998; Glendinning and Millar 1987; Millar 2007). From this perspective, poverty is less a position occupied than a condition navigated—sometimes with movement, sometimes without, often with significant effort that leaves little visible trace. Reframing poverty as lived process shifts the unit of analysis from status to trajectory. It allows attention to how constraint accumulates, how earlier experiences shape later possibilities, and how lives are oriented not only by what happens, but by what does not. Missed transitions, deferred plans, and avoided risks become analytically significant, not as absences but as outcomes of situated judgement under conditions of insecurity (Shildrick and MacDonald 2013). This perspective does not deny the importance of material resources or policy regimes; rather, it insists that their effects are realised through time, within lives that are already patterned by past exposures and future anxieties.

Such a reframing also destabilises linear assumptions about change. Poverty does not unfold as a straightforward movement between states of deprivation and security. Lives may oscillate, stall, or show fragile improvement that remains vulnerable to reversal. Understanding these patterns requires following lives longitudinally and conceptually, attending to how structural forces are lived and interpreted rather than simply recorded at moments of transition.

### 4.2 Agency as Situated, Relational, and Emotionally Mediated

Central to this ontological shift is a reconceptualisation of agency. Rather than treating agency as choice or compliance, a processual approach understands it as a situated capacity exercised within constraint, shaped by past experience, present obligation, and anticipated risk. Feminist sociology has been particularly attentive to these dimensions, showing how women's agency is embedded in care relationships, moral expectations, and institutional arrangements that both enable and limit action (Skeggs 1997; Lister 2004; Bennett 2014). From this perspective, agency is not absent in conditions of poverty, nor is it exhausted by visible movement or exit. It is expressed through endurance, conservation, recalibration, and the management of competing demands. Decisions are made not against an abstract field of options, but within emotionally charged contexts marked by fear of loss, shame, responsibility toward others, and uncertainty about the future. These emotional dynamics are not obstacles to rational action; they are integral to how judgement is formed under conditions of constraint (Reay 1996a; Walker 2014).

Recognising agency as emotionally mediated also clarifies why effort does not reliably produce change. Action oriented toward survival or stability may be entirely rational in contexts where risk carries high potential costs. Conversely, moments of reorientation often require not only material opportunity but emotional shifts—anger that disrupts accommodation, recognition that validates aspiration, or relational support that makes risk tolerable. Agency, in this sense, is neither free nor absent, but contingent and relational, exercised within open systems where outcomes cannot be guaranteed.

### 4.3 Time, Biography, and the Accumulation of Constraint

A further implication of this ontological shift is a rethinking of time. Event‐focused models privilege moments of transition because they are visible and measurable. A biographical approach, by contrast, treats time as lived duration, emphasising how experiences accumulate and sediment across lives. Life‐course scholarship has long stressed that timing matters: the same event can have different consequences depending on when it occurs, how it intersects with other domains of life, and how it is interpreted in light of past experience (Elder 1998).

In the context of poverty, this means attending to how early exposures shape later horizons, how repeated setbacks recalibrate expectations, and how prolonged insecurity narrows the range of futures that feel imaginable. Periods of apparent stasis are not empty; they are dense with activity oriented toward maintenance, care, and emotional management. Yet these forms of action remain largely invisible within theories that equate agency with transition or change. Understanding poverty through biography therefore requires analytic sensitivity to non‐events as well as events. Deferred education, avoided moves, postponed relationships, or decisions not to take risks are not simply absences; they are outcomes of accumulated constraint and emotional judgement. Bringing these dynamics into view helps explain why poverty can persist without dramatic crisis, and why change, when it occurs, is often fragile and uneven (Shildrick et al. 2017).

### 4.4 From Mis‐Specification to Reconstruction

This ontological shift also clarifies the role of structure. Structural conditions—such as labour markets, welfare regimes, housing systems, and gendered divisions of care—remain central to the production and persistence of poverty. However, rather than operating as external forces that act upon otherwise passive subjects, they are realised through lived biographies. Their effects are mediated through institutions, relationships, and emotional–relational dynamics that shape how constraint is encountered and navigated over time.

From this perspective, structure is not displaced but re‐specified. It operates through the organisation of opportunity and risk, the distribution of resources, and the production of normative expectations and forms of recognition. State action, for example, enters not only through policy design but through its lived effects: conditionality regimes that shape decision‐making, welfare encounters that produce stigma or support, and institutional arrangements that enable or constrain particular forms of action. Structure is therefore analytically inseparable from the processes through which it is lived.

This re‐specification allows for a more complete account of how poverty is reproduced. Structural conditions shape the field of possibility, but their effects are neither uniform nor immediate. They are mediated through biographies that are already patterned by past experiences and emotional orientations. In this sense, structure, agency, and emotional–relational dynamics are not separate domains, but interdependent elements of a process unfolding across time.

## 5 Poverty as Biography in Motion

Reframing poverty as a lived process requires a conceptual language capable of capturing movement, endurance, and constraint across time without reverting to static or moralised explanations. This section advances such a language through the concept of *poverty as biography in motion*. Rather than treating poverty as a status, episode, or behavioural outcome, this framework conceptualises it as an unfolding process through which lives are shaped, oriented, and reoriented under conditions of constraint. It centres biography as the site where structural forces are realised, agency is exercised, and emotion mediates what action becomes possible.

### 5.1 Biography as the Unit of Analysis

Conceptualising poverty as biography in motion shifts the unit of analysis from populations or states to lives. This move builds on narrative and life‐course sociology, which emphasise that social processes are lived and made meaningful through time, relationships, and interpretation (Elder 1998; Riessman 2003). Biographies are not simply sequences of events; they are structured by cumulative exposure, moral expectation, and emotional memory. Earlier experiences shape later judgements, not mechanically, but through the sedimentation of fear, hope, confidence, and doubt.

Approaching poverty through biography allows attention to how constraint is experienced longitudinally. Poverty is rarely encountered as a single rupture or a clearly bounded episode. Instead, it is lived as oscillation, repetition, or fragile progress, often across decades. This perspective brings into view the everyday work of managing scarcity, sustaining care, and negotiating dignity—forms of action that remain invisible within status‐based or event‐focused models. It also foregrounds the relational nature of poverty, as biographies unfold within households, communities, and institutional contexts that shape both obligation and possibility (Glendinning and Millar 1987; Dermott and Pantazis 2014). Crucially, biography in motion does not imply linear movement or inevitable progress. Lives may shift direction, stall, or circle back. What matters is not whether individuals “move out” of poverty, but how their biographies are oriented over time: toward survival, conservation, or reorientation, and under what conditions these orientations shift.

### 5.2 Trajectories as Temporal Forms of Constraint

To theorise these orientations, the framework introduces *trajectories* as temporal forms through which poverty is lived. Drawing on life‐course theory, trajectories are understood not as paths chosen freely, nor as deterministic outcomes, but as patterned processes shaped by the interaction of structure, agency, and time (Elder 1998; Millar 2007). They capture the rhythm and direction of lives under constraint, rather than discrete moments of change. Three analytically distinct but interrelated trajectory forms are particularly salient: turbulence, stalling, and fragile progress. These are not categories of people, nor stages through which all lives pass. They are temporal patterns that can coexist within a single biography and recur across different lives.

*Turbulence* refers to periods marked by instability, disruption, and rapid change, where lives are shaped by repeated crises across multiple domains—housing, work, health, relationships. In such contexts, agency is often oriented toward immediate survival, and time is experienced as compressed, uncertain, and reactive. Turbulence makes visible how structural volatility is lived and how fear and confusion shape judgement under pressure.

*Stalling* captures periods of prolonged constraint without dramatic deterioration or improvement. Lives appear static when viewed through event‐based lenses, yet stalling involves constant effort: managing scarcity, sustaining care, avoiding risk, and maintaining dignity. These periods are characterised by repetition and non‐events—opportunities deferred, moves not made—not because of passivity, but because constraint has narrowed what feels viable. Stalling foregrounds endurance as a form of action and challenges the assumption that lack of movement implies lack of agency (Shildrick and MacDonald 2013).

*Fragile progress* refers to tentative and contingent forms of reorientation, where lives begin to shift but remain vulnerable to reversal. Such progress is rarely linear or secure; it is often sustained through emotional labour, recognition, and relational support. Gains may coexist with ongoing insecurity, and the possibility of loss remains present. This trajectory highlights why “exits” from poverty are better understood as negotiated and reversible rather than definitive (Shildrick et al. 2017).

Together, these trajectory forms allow poverty to be theorised as motion without assuming progress, and as constraint without erasing agency. They make visible the temporal textures of impoverished lives and provide an analytic vocabulary for explaining persistence, endurance, and uneven change.

### 5.3 Emotion as Mediating Mechanism

Central to the concept of biography in motion is the treatment of emotion as a mediating mechanism. Emotions are not simply responses to poverty; they shape how structural conditions are interpreted, how risk is evaluated, and how action is oriented over time. Feminist and sociological research has long shown that emotions such as shame, fear, and pride are socially patterned and deeply embedded in classed and gendered relations (Reay 1996a; Skeggs 1997).

In conditions of poverty, emotion mediates agency in several key ways. Shame can narrow horizons, discouraging help‐seeking or risk‐taking; fear can orient judgement toward safety and conservation; exhaustion can limit the capacity to imagine alternative futures (Walker 2014). These emotional dynamics are not irrational barriers but rational responses to insecurity and stigma. They explain why certain actions—changing jobs, moving home, returning to education—may feel untenable even when they appear advantageous from an external perspective. Conversely, emotions can also enable reorientation. Anger may disrupt accommodation to constraint; pride and recognition can validate aspiration; hope, when supported by material and relational scaffolding, can widen what feels possible. Importantly, these emotional shifts are rarely individual achievements. They are relationally produced, emerging through encounters with institutions, communities, or significant others that recognise effort, confer legitimacy, or reduce risk (Patrick 2016).

Treating emotion as mechanism allows poverty theory to explain why agency takes different forms across trajectories. In turbulence, fear and urgency dominate; in stalling, shame and exhaustion regulate action; in fragile progress, anger and recognition can catalyse movement. Emotion thus links structure and agency across time, shaping how biographies are oriented and reoriented under constraint.

### 5.4 What This Framework Makes Visible

Conceptualising poverty as biography in motion brings into analytic focus dimensions of inequality that remain marginal within dominant approaches. It moves beyond static conceptions of poverty by foregrounding process, duration, and lived continuity. It expands the meaning of agency to include endurance, conservation, and emotional labour, rather than equating action solely with movement or exit. It also brings non‐events and periods of stalling into view, recognising them as outcomes of constraint rather than absences of action.

This perspective disrupts moralised distinctions between movement and stasis. When lives are evaluated primarily in terms of mobility or behavioural compliance, those who remain poor are easily rendered deficient. A biographical and trajectory‐based approach instead reveals how effort, judgement, and care are exercised within constrained possibilities, and why change is often fragile and reversible. At the same time, this framework remains specific to poverty. It is grounded in conditions of material constraint, institutional regulation, and unequal recognition that shape both the field of possible action and the consequences of risk. Biography in motion is not a general theory of social life, but a way of theorising how inequality is lived through time—how deprivation, insecurity, and obligation become embedded within trajectories that are difficult to shift, even in the presence of effort or opportunity. By situating structural conditions within lived biographies and treating emotional–relational dynamics as mediating mechanisms, this framework provides a theoretical grammar capable of explaining not only why poverty persists, but how it is lived, negotiated, and unevenly reconfigured across the life course.

## 6 Implications for Poverty Theory

Reconceptualising poverty as biography in motion has implications that extend beyond any single empirical setting or methodological approach. By shifting the ontological starting point from status or behaviour to lived process, this framework reshapes how poverty theory understands persistence, change, and agency, and it reframes several long‐standing debates within the field. This section outlines three core implications: for how poverty is explained, for how agency is conceptualised, and for how temporal inequality is theorised.

### 6.1 From Explanation by Condition to Explanation by Process

Much poverty theory has been oriented toward explaining *who* is poor and *why* at particular moments in time. Relative deprivation frameworks excel at identifying distributional patterns and structural drivers, whilst behavioural accounts seek to explain persistence through individual conduct. What both approaches struggle to explain is *how* poverty is lived across time—how it endures, stalls, or shifts unevenly within and across lives. A biographical and trajectory‐based approach reframes explanation as a matter of process rather than condition. Poverty is not treated as a static outcome requiring causal attribution, but as an unfolding social relation that must be followed through time. This allows theory to account for cumulative exposure, emotional sedimentation, and the ways in which past experiences shape future horizons. Persistence is no longer a residual category to be explained away, but a patterned outcome of constraint lived across biographies (Shildrick and MacDonald 2013).

This shift also alters how change is understood. Rather than assuming that movement into or out of poverty represents decisive transformation, a processual account recognises that change is often fragile, partial, and reversible. Improvement may coexist with insecurity; stability may mask ongoing vulnerability. By attending to trajectories rather than transitions alone, poverty theory gains the capacity to explain why policy interventions may register statistical effects without producing sustained biographical change (Millar 2007).

### 6.2 Re‐Specifying Agency Without Moralisation

A second implication concerns the conceptualisation of agency. Dominant poverty theories have oscillated between rendering agency invisible and inflating it in moralised terms. Structural models emphasise constraint but struggle to account for action within limits; behavioural accounts foreground choice but detach it from social context. The result is a narrow and normatively loaded understanding of agency that cannot explain endurance, risk‐aversion, or cautious adaptation. By conceptualising agency as emotionally mediated and temporally extended, the framework advanced here offers a way beyond this impasse. Agency is understood not as a moment of choice, but as an ongoing capacity to judge, adapt, conserve, and sometimes reorient within constraint. Endurance, care, and risk management become analytically significant forms of action rather than indicators of passivity or failure. This re‐specification aligns with feminist critiques of responsibilisation that emphasise the gendered and relational organisation of action under poverty (Skeggs 1997; Bennett 2014).

Crucially, this account resists moralisation. Agency is neither celebrated nor blamed; it is situated. Decisions are understood in relation to emotional economies shaped by stigma, insecurity, and obligation (Reay 1996a; Walker 2014). This allows poverty theory to explain why individuals may not pursue opportunities that appear advantageous from an external perspective, without resorting to deficit explanations. In doing so, it reclaims agency as an explanatory concept rather than a normative judgement.

### 6.3 Temporal Inequality and the Significance of Stalling

A further implication concerns how time itself is theorised in poverty research. Whilst life‐course approaches have brought attention to transitions and turning points, poverty theory has remained largely event‐focused. Lives are understood through entries and exits, employment changes, or household transitions, whilst extended periods of apparent stasis receive limited analytic attention. The trajectory‐based framework developed here foregrounds *temporal inequality*—the uneven distribution not only of resources, but of movement, security, and future possibility. Stalling, in particular, emerges as a central but under‐theorised form of poverty. Lives characterised by repetition, deferred plans, and non‐events are not empty or inactive; they are shaped by accumulated constraint and emotional regulation. Recognising stalling as a temporal form of inequality challenges assumptions that mobility is the normative or expected outcome, and it highlights how poverty can persist without dramatic crisis (Shildrick et al. 2017).

This perspective also brings non‐events into analytic focus. Decisions not to move, retrain, or leave relationships are not simply absences of action; they are outcomes of risk assessment within constrained circumstances. Attending to non‐events allows poverty theory to capture how inequality is reproduced through what does not happen as much as through what does. It also complicates celebratory narratives of resilience or mobility by showing how progress is often contingent and uneven across biographies.

### 6.4 Implications for the Scope of Poverty Theory

These implications suggest a reorientation of poverty theory away from static classifications and moralised explanations toward a more dynamic and relational account of lived inequality. Conceptualising poverty as biography in motion does not replace existing frameworks; it reframes their questions. Distributional analysis remains vital, but its findings are situated within lives that unfold over time. Behavioural insights are not dismissed, but they are reinterpreted as emotionally mediated judgements rather than as isolated choices. This reorientation also expands the scope of what poverty theory can explain. It brings into view forms of action and experience that have been marginal to dominant models: endurance without improvement, cautious adaptation, fragile reorientation, and the emotional labour of living with constraint. In doing so, it aligns poverty theory more closely with the lived realities documented by qualitative and feminist research, whilst retaining an explanatory ambition that speaks to broader sociological debates about agency, structure, and time. The concluding section draws these threads together, restating the article's central claim and reflecting on what it means to theorise poverty through lives rather than through statuses or behaviours.

## 7 Conclusion

This article has argued that a key limitation in contemporary poverty theory lies in the mis‐specification of agency. Whilst structural approaches within scholarship and behavioural framings within policy discourse differ in emphasis, both tend to rely on restricted understandings of action, rendering them less able to explain how poverty is lived across time. As a result, poverty is often conceptualised as a static condition, a sequence of discrete events, or a consequence of individual conduct, rather than as an unfolding process shaped through endurance, constraint, and mediated judgement.

In response, the article has advanced an alternative theoretical starting point: poverty as biography in motion. This reconceptualisation places lived lives at the centre of explanation, treating poverty as a temporal, relational, and emotionally mediated process rather than a fixed state or behavioural outcome. By introducing trajectories as temporal forms of constraint—turbulence, stalling, and fragile progress—it becomes possible to capture how poverty unfolds across time without assuming linear movement or definitive exit. These forms bring into analytic focus the significance of repetition, endurance, and fragility, features of poverty that have long been empirically visible but theoretically underdeveloped.

Central to this framework is the treatment of emotional–relational dynamics as mediating mechanisms. Rather than positioning shame, fear, exhaustion, or recognition as outcomes of poverty alone, the article has shown how these dynamics shape judgement, orient action, and structure what futures feel possible. This move reclaims agency as an explanatory concept without moralisation, recognising action in forms that do not conform to dominant narratives of mobility or behavioural compliance.

The contribution of this article is therefore a shift in theoretical grammar. Conceptualising poverty as biography in motion reorients explanation toward process, duration, and mediation. It invites poverty theory to follow lives rather than snapshots, to attend to non‐events as well as events, and to recognise endurance and fragility as central to how inequality is lived. In doing so, it offers a framework capable of explaining not only why poverty persists, but how it is experienced, negotiated, and unevenly reconfigured across the life course.

Understanding poverty requires more than measuring deprivation or correcting behaviour. It requires attending to how lives unfold under constraint, how agency operates within limits, and how emotional–relational dynamics shape what becomes possible over time. Taking biography seriously as a site of explanation does not dilute structural analysis; it deepens it. By following lives in motion, poverty theory can better grasp the persistence, complexity, and unevenness of inequality as it is actually lived.

## Conflicts of Interest

The author declares no conflicts of interest.

## Data Availability

The data that support the findings of this study are available on request from the corresponding author. The data are not publicly available due to privacy or ethical restrictions.
